# Prevalence of DSM-IV disorders in a population-based sample of 5- to 8-year-old children: the impact of impairment criteria

**DOI:** 10.1007/s00787-015-0684-6

**Published:** 2015-02-26

**Authors:** Jolien Rijlaarsdam, Gonneke W. J. M. Stevens, Jan van der Ende, Albert Hofman, Vincent W. V. Jaddoe, Frank C. Verhulst, Henning Tiemeier

**Affiliations:** 1 The Generation R Study Group, Erasmus MC-University Medical Center Rotterdam, Rotterdam, The Netherlands; 2 Department of Child and Adolescent Psychiatry, Erasmus MC-University Medical Center Rotterdam, Rotterdam, The Netherlands; 3 Interdisciplinary Social Sciences, Faculty of Social Sciences, University of Utrecht, Utrecht, The Netherlands; 4 Department of Epidemiology, Erasmus MC-University Medical Center Rotterdam, Rotterdam, The Netherlands; 5 Department of Paediatrics, Erasmus MC-University Medical Center Rotterdam, Rotterdam, The Netherlands; 6 Department of Psychiatry, Erasmus MC-University Medical Center Rotterdam, Rotterdam, The Netherlands

**Keywords:** Child psychiatric disorders, Prevalence, Comorbidity, Impairment, Referral for treatment

## Abstract

This study determined the impact of impairment criteria on the prevalence and patterns of comorbidity of child DSM-IV disorders. The validity of these impairment criteria was tested against different measures of mental health care referral and utilization. We interviewed parents of 1,154 children aged 5–8 years in-depth using the Diagnostic Interview Schedule for Children in Rotterdam, the Netherlands, to establish DSM-IV diagnosis. These children were randomly selected or oversampled based on Child Behavior Checklist ratings from a large population-based study (*N* = 6,172). Referral data were extracted from the psychiatric interview as well as from a follow-up questionnaire. The results showed an overall prevalence of DSM-IV disorders of 31.1 % when impairment was not considered. This rate declined to 22.9 % when mild impairment was required and declined even further, to 10.3 %, for more severe levels of impairment. Similarly, the overall comorbidity rate declined from 8.5 to 6.7 and 2.7 % when mild and severe impairment were required, respectively. Virtually all children who attained symptom thresholds for a specific disorder, and had been referred to a mental health care professional because of the associated symptoms, also had mild impairment. The requirement of severe impairment criteria significantly increased diagnostic thresholds, but for most disorders, this definition captured only half of the clinically referred cases. In conclusion, prevalence was highly dependent upon the criteria used to define impairment. If severe impairment is made a diagnostic requirement, many children with psychiatric symptoms and mild impairment seeking mental health care will be undiagnosed and possibly untreated.

## Introduction

Childhood psychiatric disorders often interfere with social and academic functioning and show considerable continuity over time [1–3]. Well-planned interventions targeting children with psychiatric disorders may serve not only to ameliorate current problems and associated impairment in functioning but also to prevent the persistence of problems into adolescence and adulthood. Epidemiological research investigating the prevalence of psychiatric disorders and patterns of comorbidity may inform nosology and intervention planning.

Despite advances in the DSM’s [4] operational definitions of disorder and psychiatric epidemiological methods, the differentiation of developmentally normal from abnormal behaviors and emotions has proven challenging [5–11]. For example, worries about losing major attachment figures or fear of dogs are common in young children and may be inappropriately diagnosed as psychiatric symptoms or disorders (e.g., separation anxiety or specific phobia). Perhaps most strikingly, structured lay interviews in epidemiological surveys have revealed unexpectedly high rates of psychiatric disorders in young children, particularly phobias [7, 8, 10]. A prime alteration in DSM-IV from DSM-III was the requirement of clinically significant distress or impairment in social, occupational, or other important areas of functioning for psychiatric diagnosis [4]. In the recently published DSM-5 [12], the criterion of clinically significant impairment or distress in functioning is retained.

While the phenomenology and symptoms of disorders were operationally defined in DSM based on expert panel consensus, impairment was left open to judgment by clinicians [5]. Despite the widespread acceptance that the requirement of clinically significant impairment helps in the differentiation from normality, there is a lack of an agreed operational definition in epidemiological studies [7, 10]. Hence, efforts to approximate the clinical diagnostic process are hampered. Specifically, there has been poor agreement between the several measures of clinical significant impairment, which has been partially attributed to the choice of thresholds (e.g., “some” or mild versus “a lot” or severe) [13]. For example, impairment questions with dichotomous (“a lot” versus “not a lot”) and scaled (“a lot,” “some,” “a little,” or “not at all”) response options have been used interchangeably [14]. It is unclear whether respondents answering “some” to the scaled question would answer “a lot” or “not a lot” to the dichotomous question [14].

Such differences (“some” versus “a lot”) in the operationalization of the DSM impairment criterion may have substantial effects on prevalence. Whereas some studies found the rate of childhood oppositional defiant disorder (ODD) to decline by approximately 38 % (from 2.9 to 1.8 %) [15], others found this rate to decline by 24 % (from 6.6 to 5.0 %) [16] and 10 % (from 13.4 to 12.1 %) [17], or to remain at the same level (8.4 %) [18]. Similarly, studies of adolescents have demonstrated substantial [19] and little [20] or no declines [21] in rates of ODD when impairment was included in the diagnostic criteria. Inconsistent patterns of findings in previous research may be explained by differences in methodologies and the lack of an accepted operational definition of the DSM impairment criterion. However, few have reported effects of multiple operationalizations of impairment on the prevalence rates of individual childhood disorders.

This study determined the 3-month prevalence and patterns of comorbidity of DSM-IV disorders in a large population-based cohort of children aged 5–8 years. We examined the impact of multiple definitions of impairment on the prevalence and comorbidity of disorders. In addition, we tested the validity of these definitions of impairment against different measures of mental health care referral and utilization. We expected prevalence to be highly dependent upon the criteria used to define impairment, with more significant decreases in rates for more severe levels of impairment. Furthermore, we expected that the addition of impairment to the diagnostic criteria serves not only as a way to increase thresholds, but may also more accurately indicate clinically referred cases.

## Method

### Sample and procedure

This research was embedded in the Generation R Study, a population-based cohort from fetal life onward. Sample ascertainment and participation have been described in detail elsewhere [22]. Pregnant women living in the study area in Rotterdam, the Netherlands, with an expected delivery date between April 2002 and January 2006 were invited to participate. In this paper, we utilize data obtained as part of a Generation R early school age (5–9 years) follow-up study [22]. The study was conducted in accordance with the guidelines proposed in the World Medical Association Declaration of Helsinki and was approved by the Medical Ethical Committee of the Erasmus University Medical Center, Rotterdam. Written consent was obtained from all participants.

In the Generation R Study, a total of 9,276 children were invited for follow-up assessments at early school age, and 8,305 of these children participated. For the purpose of efficient estimation of prevalence rates of psychiatric disorders, we focused on a subsample enriched for psychopathology and employed a two-stage design. We defined cut-off points based on the Child Behavior Checklist preschool form (CBCL 1.5/5) [23] total problems score (the top 15 percent) and the syndrome scale scores (the top 2 percent) to identify children with a high probability of disorder. The psychometric properties of the CBCL are well established [23].

The CBCL preschool form (age 1.5–5) as well as the CBCL school form (age 6–18) could have been applied to children in the age range of 5–7 years. Because we anticipated the majority of children (59 % in this sample) to be younger than 6 years at the time of assessment, and assessment waves relied on sending the parents of children the same questionnaire, we decided to use the CBCL preschool form for all children. CBCL data were available for 6,252 children, 75 % of those eligible (*N* = 8,305) and 67 % of those in the original cohort (*N* = 9,276). Non-respondents in the original cohort (*N* = 3,024) did not differ from respondents (*N* = 6,252) in terms of gender. However, non-respondents were less often Dutch (39 versus 61 %, χ^2^(1) = 369.26, *p* < 0.001) and their parents were more often single (29 versus 14 %, χ^2^(1) = 16.67, *p* < 0.001) and lower educated (χ^2^(2) = 51.93, *p* < 0.001). We excluded 80 children aged ≥8 years, leaving a sample of 6,172 children (mean age = 6.03, SD = 0.40) eligible for in-depth diagnostic assessment.

Given our focus on a subsample of children enriched for psychopathology, all children scoring above the predetermined cut-off points (screen-positives, *N* = 1,080), and a random selection of children scoring below the cut-off points (screen-negatives, *N* = 330) were recruited for in-depth interview. These 330 selected screen-negative children did not differ from the non-selected screen-negative children in terms of child gender, national origin, parental marital status, and maternal educational level (all *p* > 0.05). Parents of the total of 1,410 targeted children were contacted by telephone to make an appointment for a home visit. If parents were not reached after at least eight attempts, contact was made by mail or by personal visits. No informant could be reached for 102 of these 1,410 children. Interview data were obtained from 1,166 participants, 89 % of those reached. Non-respondents (*N* = 142) did not differ from respondents (*N* = 1,166) in terms of gender and parental marital status. However, non-respondents were less often Dutch (45 versus 55 %, χ^2^(1) = 4.65, *p* = 0.031) and their mothers were lower educated (χ^2^(2) = 8.42, *p* = 0.015). Twelve children were older than 9 years at the time of interview and were excluded from analyses. This left 1,154 children with interview data (mean age = 6.74, SD = 0.59) in our full sample (*N* = 6,172).

### Psychiatric interview

The diagnostic interview schedule for children-young child version (DISC-YC) [24] is a highly structured DSM-IV [4]-based interview to be administered to the parents or caregivers of children aged 3–8 years. The DISC-YC is a developmentally appropriate adaptation of the DISC-parent version (DISC-P; intended for children aged 6–17 years). Building on the well-validated DISC-P with only minor developmentally specific adaptations including the timeframe (generally the past 3 months instead of the past year) and question wording (e.g., “other children” instead of “other people”), the DISC-YC was nevertheless subjected to psychometric testing. Data on the acceptable to high overall reliability of the DISC-YC symptom scales have not been formally published but were obtained through personal communication with the authors and were also reported by others (test–retest reliability for attention-deficit/hyperactivity disorder (ADHD) scales = 0.67, for ODD = 0.88, for anxiety and depression scales = 0.57–0.81) [17]. The timeframe of the DISC-YC for determining the presence of disorders is generally the past 3 months. Only for dysthymia and conduct disorder a 1-year timeframe was used.

We used the computer-assisted DISC-YC, which is programmed to derive DSM-IV diagnoses by applying the algorithms provided by the developers. The DISC-P had previously been translated into Dutch and verified by back translation into English. Two research staff members translated the parts of the DISC-YC that had been adapted to be developmentally appropriate. Subsequently, a third research staff member involved in the DISC-P translation reviewed and approved their translations.

Like the DISC-P, the DISC-YC is designed to be administered by lay interviewers. Six interviewers were trained to administer the DISC-YC during an official training program held by the DISC-YC staff. Bilingual interviewers (Dutch and Turkish, Dutch and Arabic or Dutch and Amazigh (Berber)) were trained to interview parents who were not sufficiently fluent in Dutch to participate. Although the interviewers were informed of the study purposes, they were blind to the CBCL groupings (scores below or above cut-off points).

*Impairment.* At the end of each diagnostic section in the DISC-YC, there are 6-stem/contingent question pairs to assess “impairment” from the symptoms of the disorder. These DISC-YC questions on impairment include whether the symptoms reported (1) caused caretakers to be annoyed or upset, (2) prevented the child from doing things or going places with family, (3) prevented the child from doing things or going places with other children, (4) caused problems with activities, tasks, or play, (5) caused teachers or additional caretakers to be annoyed or upset, and (6) caused distress to the child. These impairments are rated on a three-point scale: “a lot of the time,” “some of the time,” or “hardly ever” (or in the case of problems with activities: “very bad,” “bad,” or “not too bad”).

The current study reports prevalence rates at three levels: (1) “all children meeting the DSM-IV symptom criteria” including all children displaying the minimum number of symptoms needed for diagnosis, (2) “at least mild impairment” imposing the requirement that at least one impairment has been given an “intermediate” or “severe” rating (i.e., “some of the time,” “a lot of the time,” “bad,” or “very bad”), and (3) “severe impairment” imposing the requirement that at least one impairment has been given a “severe” rating (i.e., “a lot of the time” or “very bad”).

The multiple definitions of impairment were first validated against the DISC-YC items on mental health care referral and utilization. At the end of each diagnostic section in the DISC-YC, parents were asked whether or not the child had been to someone at a hospital, a clinic or other mental health institute in the last three months because of the symptoms. The impairment definitions were then validated against referral data collected in the whole Generation R cohort as part of a subsequent assessment wave at age 8 years (*N* = 4,399 in the full sample; *N* = 795 in the DISC-YC sample). Parents rated whether or not the child had been referred to an in- or out-patient clinic in the last year as a result of anxiety, mood, ADHD, or disruptive behavior problems.

### Statistical analysis

Statistical analyses were carried out using the computer package SPSS Complex Samples Statistics (IBM SPSS Statistics version 20.0). This procedure takes account of the complex survey design and applies weights to adjust for unequal sampling probabilities to represent rates of disorder in the full sample (*N* = 6,172). Specifically, we restricted the analyses to those 1,154 children with interview data and assigned a sampling weight to each child, representing the inverse of the stage two sampling fraction (i.e., screen-positives in stage one/screen-positives in stage two; screen-negatives in stage one/screen-negatives in stage two) [25].

The 3-month prevalence of the DISC-YC-derived diagnoses was determined without and with the additional impairment criteria. Logistic regression analysis was used to examine the associations of child gender with child psychiatric disorder and the odds of comorbidity between pairs of diagnoses. When low rates of individual diagnoses precluded meaningful logistic regression analysis, DSM-IV diagnoses were collapsed into the following diagnostic groupings [19]: anxiety, mood, ADHD, and conduct/oppositional disorder. Comorbidity between ODD and conduct disorder could not occur because, according to the DSM-IV criteria, a diagnosis of conduct disorder takes precedence over a diagnosis of ODD.

Validity testing of the impairment criteria was performed in two steps. We first used the referral data extracted from the DISC-YC and examined the extent to which children who attained symptom thresholds for a specific disorder, and had been referred as a result of the symptoms, also had mild or severe impairment. We then used the referral data collected as part of the subsequent data wave at age 8 years. We examined the weighted prevalence of referral to an in- or out-patient clinic at age 8 years per DISC-based diagnostic grouping and per level of impairment.

## Results

### Prevalence of DSM-IV disorders

Table 1 reports the 3-month prevalence of DSM-IV disorders in selected 5- to 8-year-old children (*N* = 1,154) weighted to represent rates in the full sample (*N* = 6,172). Rates of disorder are presented according to level of impairment required for diagnosis. The children who had data on the DISC-YC at age 6 years (i.e., weighted subsample) were representative of the full sample in terms of gender (52 versus 50 % were boys, respectively), national origin (64 versus 62 % were Dutch, respectively), maternal education (10 versus 12 % attained ≤3 years general secondary school, respectively), and parental marital status (10 % were single).

**Table 1:** Three-month prevalence of DSM-IV disorders among 5- to 8-year-olds according to level of impairment required for diagnosis (*N* = 6,172)

Diagnosis	Impairment not required		Mild impairment required		Severe impairment required	
	%	(95 % CI)	%	(95 % CI)	%	(95 % CI)
Any disorder	31.1	(26.9–35.6)	22.9^a^	(19.3–26.9)	10.3^b,c^	(7.9–13.2)
Anxiety disorders						
Any anxiety disorder	12.7	(9.9–16.1)	7.8^a^	(5.7–10.7)	2.4^b,c^	(1.4–4.1)
Social phobia	1.4	(0.7–2.5)	1.2	(0.6–2.4)	0.6	(0.2–1.6)
Separation anxiety	1.3	(0.6–2.8)	0.9	(0.4–2.2)	0.3^b,c^	(0.1–0.5)
Specific phobia	10.7	(8.1–14.0)	6.1^a^	(4.1–8.8)	1.6^b,c^	(0.8–3.3)
Generalized anxiety disorder	0.5	(0.3–0.7)	0.4	(0.3–0.7)	0.1^b,c^	(0.0–0.3)
Obsessive–compulsive disorder	0.5	(0.1–1.6)	0.2	(0.1–0.3)	0.1	(0.0–0.2)
Post-traumatic stress disorder	0.1	(0.0–0.2)	0.1	(0.0–0.2)	0.0	(0.0–0.2)
Mood disorders						
Any mood disorder	0.8	(0.3–2.2)	0.8	(0.3–2.2)	0.4	(0.1–1.6)
Major depressive episode	0.4	(0.1–1.8)	0.4	(0.1–1.8)	0.3	(0.1–1.9)
Dysthymia	0.5	(0.1–1.6)	0.4	(0.1–1.7)	0.1	(0.0–0.3)
Behavioral disorders						
Any behavioral disorder	16.4	(13.4–19.9)	15.5	(12.6–18.9)	8.1^b,c^	(6.1–10.7)
Any ADHD	8.7	(6.6–11.3)	8.0	(6.1–10.6)	4.6^b,c^	(3.2–6.7)
ADHD-inattention	3.2	(2.0–5.2)	2.7	(1.6–4.6)	1.4	(0.7–2.9)
ADHD-hyperactive	2.8	(1.8–4.5)	2.7	(1.7–4.4)	1.6	(0.8–3.0)
ADHD-combined	2.6	(1.7–4.1)	2.6	(1.7–4.1)	1.7	(0.9–3.1)
Oppositional defiant disorder	10.5	(8.1–13.5)	10.1	(7.8–13.1)	4.6^b,c^	(3.2–6.7)
Conduct disorder	0.3	(0.2–0.5)	0.2	(0.1–0.4)	0.1	(0.0–0.3)
Miscellaneous						
Nocturnal enuresis	6.6	(4.6–9.2)	2.2^a^	(1.2–3.9)	0.2^b,c^	(0.1–0.4)
Diurnal enuresis	0.7	(0.3–1.7)	0.6	(0.2–1.6)	0.1^b,c^	(0.0–0.2)
Encopresis	1.5	(0.7–3.2)	1.2	(0.5–2.7)	0.7	(0.2–2.2)
Tourette’s disorder	0.0	(0.0–0.2)	0.0	(0.0–0.2)	0.0	(0.0–0.2)

Weighted prevalence estimates (95 % confidence intervals) based on Diagnostic Interview Schedule for Children (DISC) interviews in 1,154 selected children to represent rates in the full population-based sample (N = 6,172). The timeframe of the DISC for determining the presence of disorders was generally the past 3 months. Only for dysthymia and conduct disorder a 1-year timeframe was used

*ADHD* attention-deficit/hyperactivity disorder

^a^Decrease in rate is statistically significant: mild impairment required versus impairment not required

^b^Decrease in rate is statistically significant: severe impairment required versus impairment not required

^c^Decrease in rate is statistically significant: severe impairment versus mild impairment required

Of the children in this sample, 31.1 % attained DSM-IV symptom thresholds for any disorder. The two most prevalent disorders were ODD (10.5 %) and specific phobia (10.7 %), followed by ADHD (8.7 %). Within the ADHD category, the rates for the inattentive type, the hyperactive type, and the combined type were 3.2, 2.8, and 2.6 %, respectively. The overall prevalence of 31.1 % declined to 22.9 % when mild impairment was required and declined even further, to 10.3 %, for more severe levels of impairment. We compared prevalence rates and their 84 % confidence intervals to detect change due to a more stringent case definition (see also Table 1) [26]. Whereas rates of behavioral disorders and mood disorders remained largely unchanged when mild impairment was added to the diagnostic criteria, rates of anxiety disorders, and in particular specific phobia, declined considerably. When severe impairment was required, prevalence rates significantly declined for both anxiety (from 12.7 to 2.4 %) and behavioral (from 16.4 to 8.1 %) disorders.

Table 2 shows prevalence estimates for DSM-IV diagnostic groupings by child gender. As shown in Table 2, none of the associations with child gender reached statistical significance in logistic regression analysis. However, a marginally significant gender difference was found in the occurrence of any disorder without the requirement of impairment (35.3 % in boys versus 26.5 % in girls, OR = 0.66, 95 % CI = 0.44–1.00).

**Table 2:** Three-month prevalence estimates for DSM-IV diagnostic groupings according to level of impairment required for diagnosis and gender

Diagnostic grouping	Boys (*N* = 637) % (95 % CI)	Girls (*N* = 517) % (95 % CI)	OR (95 % CI)
Any disorder, impairment not required	35.3 (29.3–41.7)	26.5 (20.9–33.0)	0.66 (0.44–1.00)
Any disorder with mild impairment	25.6 (20.5–31.4)	19.9 (15.1–25.8)	0.72 (0.46–1.12)
Any disorder with severe impairment	10.7 (7.7–14.7)	9.7 (6.5–14.4)	0.90 (0.51–1.59)
Anxiety, impairment not required	12.4 (8.8–17.3)	13.0 (9.0–18.3)	1.05 (0.60–1.83)
Anxiety with mild impairment	8.2 (5.4–12.3)	7.5 (4.6–11.9)	0.90 (0.46–1.79)
Anxiety with severe impairment	3.3 (1.7–6.4)	1.4 (0.6–3.4)	0.41 (0.13–1.28)
Mood, impairment not required	0.8 (0.2–3.1)	0.8 (0.2–3.6)	0.94 (0.12–7.17)
Mood with mild impairment	0.8 (0.2–3.2)	0.8 (0.2–3.6)	0.99 (0.12–7.86)
Mood with severe impairment	0.2 (0.1–0.5)	0.7 (0.1–3.8)	3.45 (0.50–24.04)
ADHD, impairment not required	10.3 (7.4–14.1)	7.0 (4.4–10.9)	0.65 (0.36–1.20)
ADHD with mild impairment	9.2 (6.5–12.9)	6.8 (4.2–10.7)	0.72 (0.38–1.34)
ADHD with severe impairment	4.9 (3.1–7.8)	4.3 (2.3–7.9)	0.86 (0.38–1.95)
Conduct/oppositional, impairment not required	12.9 (9.4–17.5)	8.4 (5.5–12.8)	0.62 (0.35–1.12)
Conduct/oppositional with mild impairment	12.2 (8.8–16.6)	8.3 (5.4–12.6)	0.65 (0.36–1.18)
Conduct/oppositional with severe impairment	5.5 (3.5–8.7)	3.9 (2.1–7.2)	0.69 (0.31–1.55)

Weighted prevalence estimates (95 % confidence intervals) based on Diagnostic Interview Schedule for Children (DISC) interviews in 1,154 selected 5- to 8-year-olds to represent rates in the full population-based sample (*N* = 6,172)

*ADHD* attention-deficit/hyperactivity disorder

### Impairment

We first validated the impairment criteria against DISC-based, diagnosis-specific referral rates. All children (or all except one for social phobia, generalized anxiety, ADHD, and conduct disorder) who attained symptom thresholds for a specific disorder, and had visited a mental health care professional because of the associated symptoms, also had mild impairment. In contrast, approximately half of the children who attained symptom thresholds and had visited a mental health care professional because of symptoms associated with social phobia, generalized anxiety, ADHD and ODD, also had severe impairment. Only for separation anxiety, all children who attained symptom thresholds for disorder, and had been referred for treatment, also had severe impairment.

Next, we examined the weighted prevalence of referral at age 8 years per DISC-based diagnostic grouping and per level of impairment. Table 3 shows that for all diagnostic groupings the weighted prevalence of referral was consistently low in children falling below DISC symptom thresholds for diagnosis (range = 1.3–2.2 %). As expected, these rates of referral were higher in those children who attained symptom thresholds for mood (44 %), ADHD (26.8 %), and conduct/oppositional (15.1 %) disorders. Furthermore, these rates of referral were higher in those children who attained symptom thresholds with mild (range = 15.3–45.5 %) and severe (range = 24.0–82.0 %) impairment. Only few children attaining symptom thresholds for anxiety disorders, with or without the requirement of impairment, were referred (e.g., rate of referral was 4.9 % when severe impairment was required).

**Table 3:** Prevalence of mental health care referral and utilization at age 8 years per DSM-IV diagnostic grouping and level of impairment (*N* = 6,172)

Diagnostic groupings	Mental health care referral and utilization % (95 % CI)
Anxiety	
Below symptom threshold	2.2 (1.0–4.8)
Above symptom threshold	
Impairment not required	2.0 (0.9–4.1)
Mild impairment required	3.1 (1.4–6.8)
Severe impairment required	4.9 (1.7–13.7)
Mood	
Below symptom threshold	1.9 (0.8–4.2)
Above symptom threshold	
Impairment not required	44.0 (5.0–92.1)
Mild impairment required	45.4 (4.8–93.2)
Severe impairment required	82.0 (19.7–98.8)
ADHD	
Below symptom threshold	1.3 (0.6–2.6)
Above symptom threshold	
Impairment not required	26.8 (15.3–42.7)
Mild impairment required	27.8 (15.6–44.5)
Severe impairment required	37.8 (18.6–61.8)
Conduct/oppositional	
Below symptom threshold	1.4 (0.5–3.4)
Above symptom threshold	
Impairment not required	15.1 (6.9–29.8)
Mild impairment required	15.3 (7.0–30.1)
Severe impairment required	24.0 (9.6–48.4)

Weighted prevalence estimates (95 % confidence intervals) based on Diagnostic Interview Schedule for Children (DISC) interviews in 1,154 selected children to represent rates in the full population-based sample (*N* = 6,172)

*ADHD* attention-deficit/hyperactivity disorder

### Comorbidity of DSM-IV disorders

The prevalence of general comorbidity (i.e., two or more DSM-IV disorders of any type) was 8.5 % when impairment was not considered, and decreased to 6.7 and 2.7 % when mild and severe impairment criteria were employed, respectively. The odds of comorbidity between pairs of diagnoses were examined using logistic regression analysis. Table 4 shows that when impairment was not considered, ADHD was associated with anxiety (OR = 2.62, 95 % CI = 1.36–5.07) and conduct/oppositional disorders (OR = 5.97, 95 % CI = 3.15–11.3). When mild impairment was required, the associations between disorders were generally large and statistically significant, with the exception that mood disorders were unrelated to all other disorders (see Table 4). That is, ADHD was associated with conduct/oppositional disorder (OR = 6.44, 95 % CI = 3.32–12.5) and anxiety disorders (OR = 4.17, 95 % CI = 1.94–8.97). Furthermore, anxiety disorders were associated with conduct/oppositional disorder (OR = 2.78 (95 % CI = 1.28–6.01). We additionally examined the association between disorders imposing a requirement of severe impairment. Again, ADHD was associated with conduct/oppositional disorder (OR = 9.69, 95 % CI = 3.80–24.7) and anxiety disorders (OR = 7.19, 95 % CI = 1.98–26.13). Also, anxiety disorders were associated with conduct/oppositional disorder (OR = 7.12, 95 % CI = 1.95–26.0) and mood disorder (OR = 10.28, 95 % CI = 1.41–74.68).

**Table 4:** Odds ratios for comorbidity between four DSM-IV diagnostic groupings among 5- to 8-year-old children (*N* = 6,172)

Diagnostic groupings	Anxiety		Mood		ADHD		Conduct/oppositional	
	OR	(95 % CI)	OR	(95 % CI)	OR	(95 % CI)	OR	(95 % CI)
Anxiety	–	–	1.55	(0.36–6.63)	2.62	(1.36–5.07)	1.83	(0.95–3.53)
Mood	**2.29**	**(0.51–10.2)**	–	–	2.38	(0.55–10.2)	2.97	(0.66–13.4)
ADHD	**4.17**	**(1.94–8.97)**	**2.21**	**(0.50–9.80)**	–	–	5.97	(3.15–11.3)
Conduct/oppositional	**2.78**	**(1.28–6.01)**	**2.80**	**(0.61–12.8)**	**6.44**	**(3.32–12.5)**	–	**–**

Weighted odds ratios (ORs) for comorbidity between disorders based on Diagnostic Interview Schedule for Children (DISC) interviews in 1,154 selected children to represent odds ratios in the full population-based sample (*N* = 6,172). CI indicates confidence interval. Upper triangular area values represent odds ratios for disorders without the requirement of impairment and lower triangular values (boldface) represent odds ratios for disorders with mild impairment

*ADHD* attention-deficit/hyperactivity disorder

## Discussion

In this population-based cohort, an estimated 31.1 and 22.9 % of 5- to 8-year-old children had at least one psychiatric disorder without and with the additional requirement of impairment, respectively. This rate declined to 10.3 % when more severe levels of impairment were required for diagnosis. The prevalence of general comorbidity was 8.5 % when impairment was not considered, and declined to 6.7 and 2.7 % when mild and severe impairment were required for diagnosis, respectively.

The overall prevalence of psychiatric disorders with mild impairment is consistent with previous reports of young, school-age children [16, 18, 27]. The most common disorders were behavioral disorders, particularly ODD. Behavioral disorders without the consideration of impairment occurred in 16.4 % of our sample, which resembled the 15.9 % observed by McArdle et al. [16] and the 14.3 % observed by Carter et al. [18], who also investigated the impact of impairment criteria on rates of disorder [16, 18]. Whereas McArdle et al. [16] found the rate of behavioral disorders to decline by 34 % (to 10.5 %) when moderate impairment was required for diagnosis, the current study and the Carter et al. [18] study found this rate to remain largely unaltered.

The lack of a clear pattern in the impact of impairment criteria may be attributable to the considerable variation in the operationalizations or measurements of functional impairment. For example, McArdle et al. [16] used a measure of global impairment, whereas the current study and the Carter et al. [18] study used DISC-based diagnosis-specific impairment measures. There has been poor agreement between these two measures of impairment, which has been partially attributed to the choice of thresholds (e.g., mild or moderate versus “a lot” or severe) [13]. However, few studies have reported effects of multiple operationalizations of impairment on the prevalence of childhood disorders. We showed that the requirement that the symptoms interfered with children’s functioning “a lot of the time” (conceptualized here as severe impairment) had a significantly greater impact on the rates of anxiety and behavioral disorders than the requirement that the symptoms interfered with children’s functioning “some of the time” (conceptualized here as mild impairment), as indicated by the non-overlapping confidence intervals.

Depending upon the type of disorder, a considerable amount of children who attained symptom thresholds for diagnosis appeared not to show impairment. This was most marked in the case of specific phobia, which, as has been found in other population-based studies [15, 18, 20], was very common when symptom thresholds alone were considered. However, specific phobia was much less common when mild impairment was required for diagnosis. Conversely, rates of behavior problems decreased considerably only when a more severe degree of impairment was required for diagnosis. The DSM-IV symptom criteria of behavioral diagnoses often require associated impairment at a clinically significant level (e.g., aggression toward other people). Thus, many children who attain symptoms thresholds for diagnosis will also satisfy the impairment criterion [6]. Similarly, for depressive disorders, whose symptoms may be considered to be intrinsically impairing (e.g., markedly diminished interest or pleasure in activities), the requirement of impairment had little or no effect on prevalence [15, 17–21, 28]. One may argue, like Rutter [10] and Spitzer and Wakefield [6], that impairment can be better captured by the modification of symptom criteria. For example, Rutter proposed that “in order to deal with the ‘over-diagnosis’ of phobias problem, a simple remedy would be to treat avoidance as a symptom rather than an impairment” [10].

One of the few longitudinal studies of child psychiatric disorders beginning in the preschool years showed that the stability of diagnosis was high for behavioral disorders but only moderate for emotional disorders [3]. For example, depressive disorders are substantially more common among adolescents and adults than among young children [29], and adult symptom criteria will less likely identify a valid childhood psychiatric disorder that can be reliably measured using structured diagnostic interviews [30]. Conversely, worries about losing major attachment figures or fear of dogs are substantially more common at younger than at older ages, and rates of separation anxiety disorder or specific phobia diagnosed using developmentally inappropriate criteria may be invalid. Future research is needed to understand the relationships between psychiatric symptoms, different levels of impairment, and diagnosis over time.

Given the structure of the DISC interviews, impairment ratings were obtained only if a certain amount of symptoms was present. It is well established that many children attaining diagnostic thresholds are little impaired, whereas, although not testable here, many children with subthreshold disorders may suffer from associated impairment [20, 28, 31]. Pickles et al. [28] showed that mood and behavioral disorders differ considerably in the extent to which impairment is prognostic. For behavioral disorders, both symptoms and impairment at age 8–16 years were associated with symptoms, diagnosis, and impairment 1.5 years later. For mood disorders, only current symptoms and not impairment scores were associated with future symptoms, diagnosis, and impairment [28]. The relative importance of symptom and impairment criteria for psychiatric classification and clinical decision-making warrants further investigation. We showed that, in addition to symptom criteria, the mild impairment criterion captured virtually all clinically referred cases. However, for most disorders, the severe impairment criterion captured only half of the clinically referred cases.

Although odds ratios for comorbidity increased with the addition of impairment to the diagnostic criteria, confidence intervals were overlapping. Therefore, it cannot be concluded that comorbidity rates differ depending on whether impairment is required or not. The co-occurrence of two or more DSM-IV disorders was common. The current findings concur with those of other investigators [20, 27, 32], indicating that comorbidity is evident among emotional or behavioral disorders, but also across these domains of disorder. Consistent with previous research [15], we observed a marginal gender difference in the prevalence of any psychiatric disorder, with boys outnumbering girls.

Next to the various strengths of our study, including the use of a large prospective population-based cohort, and the collection of data through structured diagnostic interviews, several limitations should be considered. The fact that the current study was conducted in a diverse, urban area is certainly an advantage but does not necessarily imply that findings are easily generalizable to the larger population of children and their families. As in many prospective cohort studies, attrition is a concern. Furthermore, despite being considered an efficient means for estimating prevalence of psychiatric disorders that are relatively uncommon, a two-stage sampling design may reduce statistical precision [25, 29]. It has been recommended to use multiple reporters in the assessment of outcome when employing a two-stage design [29]. If anything, our use of a single parent informant may have led to an underestimation of the potentially more subtle impairments of children with anxiety disorders versus disruptive disorders. The fact that mental health treatment for children is often initiated by adults, and the possibility that impairment brings symptomatology to the attention of adults [31], may explain the relatively low rates of referral in children with anxiety disorders. However, because of children’s young age (5–8 years), children’s self-reports of disorders and associated impairment on the DISC would have likely been unreliable [33, 34]. Clearly, a diagnosis based on clinicians’ ratings would have been a valuable but costly addition to the structured interviews. Finally, given that assessment waves relied on sending children in a small age range (5–7 years) the same screening questionnaire, we also used the data of those children (41 %) who fell outside the age range for which the questionnaire was designed (age 1.5–5 years). However, tests of internal consistency have indicated that behavioral and emotional problems were also reliably measured in children older than 5 years [35].

In conclusion, findings from this large population-based cohort of 5- to 8-year-old children showed that prevalence was highly dependent upon the criteria used to define impairment. Virtually all children who attained symptom thresholds for a specific disorder, and had been referred to a mental health care professional because of the associated symptoms, also had mild impairment. The requirement of severe impairment criteria significantly increased diagnostic thresholds but for most disorders this definition captured only half of the clinically referred cases. According to the observed mental health care referral and utilization patterns, not only those children with severe levels of impairment, but also those with milder levels should be considered to meet criterion. Findings of this study suggest that if severe impairment is made a diagnostic requirement, many children with psychiatric symptoms and mild impairment seeking mental health care will be undiagnosed and possibly untreated.
